# Amyloid-Beta_1-42_ -Induced Increase in GABAergic Tonic Conductance in Mouse Hippocampal CA1 Pyramidal Cells

**DOI:** 10.3390/molecules25030693

**Published:** 2020-02-06

**Authors:** Beatriz Calvo-Flores Guzmán, SooHyun Kim, Bhavya Chawdhary, Katie Peppercorn, Warren P Tate, Henry J Waldvogel, Richard LM Faull, Johanna Montgomery, Andrea Kwakowsky

**Affiliations:** 1 Centre for Brain Research, Department of Anatomy and Medical Imaging, Faculty of Medical and Health Sciences, University of Auckland, Auckland 1023, New Zealand; b.guzman@auckland.ac.nz (B.C.-F.G.); soo.kim@auckland.ac.nz (S.K.); b.chawdhary@auckland.ac.nz (B.C.); h.waldvogel@auckland.ac.nz (H.J.W.); rlm.faull@auckland.ac.nz (R.L.F.); 2 Department of Biochemistry, University of Otago, Dunedin 9054, New Zealand; katie.peppercorn@otago.ac.nz (K.P.); warren.tate@otago.ac.nz (W.P.T.); 3 Centre for Brain Research, Department of Physiology, Faculty of Medical and Health Sciences, University of Auckland, Auckland 1023, New Zealand; jm.montgomery@auckland.ac.nz

**Keywords:** GABA 1, GABA_A_ receptor 2, tonic inhibition 3, amyloid-beta 4, hippocampus 5

## Abstract

Alzheimer’s disease (AD) is a complex and chronic neurodegenerative disorder that involves a progressive and severe decline in cognition and memory. During the last few decades a considerable amount of research has been done in order to better understand tau-pathology, inflammatory activity and neuronal synapse loss in AD, all of them contributing to cognitive decline. Early hippocampal network dysfunction is one of the main factors associated with cognitive decline in AD. Much has been published about amyloid-beta_1-42_ (Aβ_1-42_)-mediated excitotoxicity in AD. However, increasing evidence demonstrates that the remodeling of the inhibitory gamma-aminobutyric acid (GABAergic) system contributes to the excitatory/inhibitory (E/I) disruption in the AD hippocampus, but the underlying mechanisms are not well understood. In the present study, we show that hippocampal injection of Aβ_1-42_ is sufficient to induce cognitive deficits 7 days post-injection. We demonstrate using *in vitro* whole-cell patch-clamping an increased inhibitory GABAergic tonic conductance mediated by extrasynaptic type A GABA receptors (GABA_A_Rs), recorded in the CA1 region of the mouse hippocampus following Aβ_1-42_ micro injection. Such alterations in GABA neurotransmission and/or inhibitory GABA_A_Rs could have a significant impact on both hippocampal structure and function, causing E/I balance disruption and potentially contributing to cognitive deficits in AD.

## 1. Introduction

The role of the inhibitory gamma-aminobutyric acid (GABA) system in excitatory/inhibitory (E/I) balance disruption in the Alzheimer’s disease (AD) brain has been described to occur before, and to possibly underlie, amyloid-beta (Aβ) mediated-excitotoxicity. Despite that GABAergic neurons seem to be relatively spared during AD progression, there is evidence that demonstrates that they are vulnerable to exposure to Aβ neurotoxic protein fragments [1]. For example, GABAergic parvalbumin (PV)-positive interneurons that are inhibitory and fast spiking, key regulators of E/I balance through the synchronization of excitatory neuronal network activity in the cortex and in the hippocampus [2], have been shown to be impaired by Aβ, ultimately causing the impairment of inhibitory function, disrupting of E/I balance and causing cognitive decline in AD [3]. 

Increases in ambient GABA levels may also be involved in learning and memory dysfunction in AD [4]. Deficits in mitochondrial ATP occurring with ageing promotes increased ambient GABA levels through release of GABA in the extrasynaptic space [5]. However, Aβ may also be indirectly involved in the release of ambient GABA by astrocytes to the extrasynaptic space [6,7]. In the hippocampus, ambient GABA levels activate extrasynaptic α5 subunit-containing type A GABA receptors (GABA_A_Rs), mostly expressed in the somatodendritic region of CA1 pyramidal layer of the hippocampus, where they mediate tonic inhibition [8,9]. Persistent and continuous activation of these receptors due to increased ambient GABA levels generates chronic hyperpolarizing conductance, ultimately affecting the excitability of neurons in the hippocampus [10], thus contributing to the alteration of the E/I balance. 

Restoration of E/I balance could reverse neuronal dysfunction, contributing to the amelioration of cognitive decline in AD [11,12]. Given the potentially promising pharmacological advances in this direction [13,14,15], we aimed to assess Aβ_1-42_-induced changes in the functional activity of the GABA_A_Rs in CA1 pyramidal cells. 

## 2. Results and Discussion

**Figure 1:**
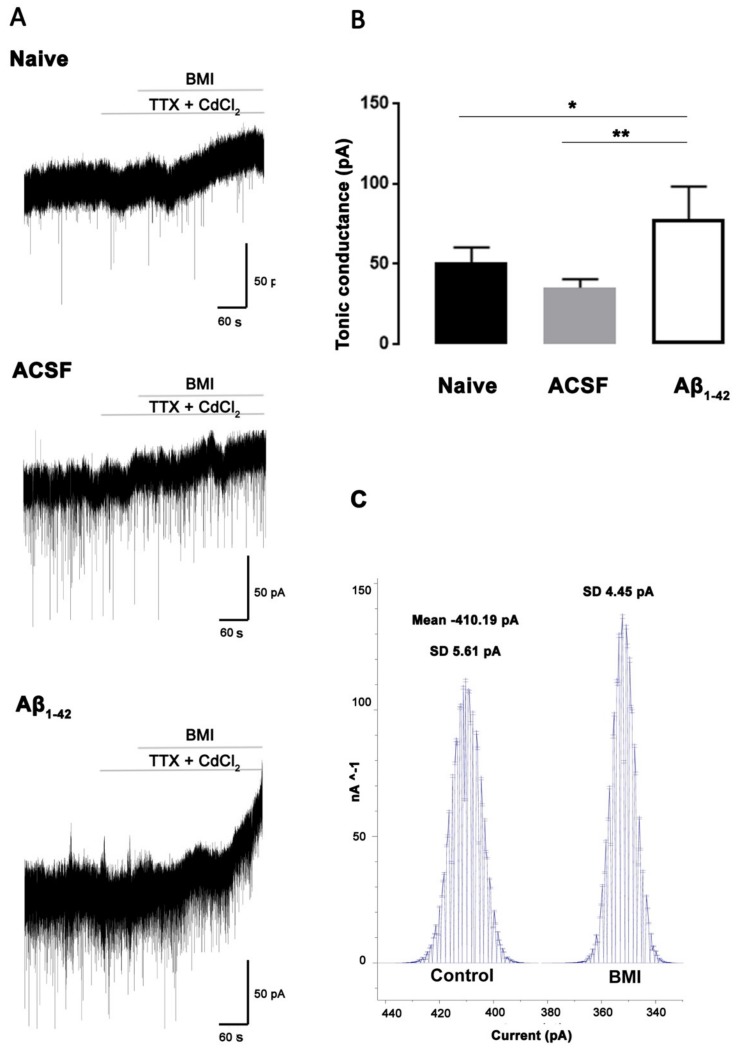
Aβ_1-42_-induced increase in gamma-aminobutyric acid (GABAergic) tonic conductance in CA1 pyramidal cells. (**A**) Representative whole cell traces from voltage-clamp recording of CA1 pyramidal cells (PCs) (Vh = −70 mV) from naïve control (NC), artificial cerebrospinal fluid (ACSF) -injected and Aβ_1-42_-injected mice in the presence of 3 mM kynurenic acid and 5 µM GABA. The lines above the traces indicate the points at which TTX (5 µM) + CdCl_2_ (50 µM) and bicuculline methiodide (BMI) (100 µM) were applied. (**B**) Histogram plot of tonic current measured in the NC, ACSF-injected and Aβ_1-42_-injected mice. Data are expressed as mean ± SD (one-way ANOVA and Tukey’s post-hoc test (* *p* = 0.0382, ** *p* = 0.0025; NC *n* = 4, ACSF-injected *n* = 4, and Aβ_1-42_-injected *n* = 5). (**C**) For a given CA1 PC (in this case from a NC), tonic current was determined by generating all-points histograms for the control period (before BMI application) and during the BMI application period, corresponding to 60 s per period (600001 points each). Mean values from each histogram were used to determine the current during the control and BMI periods. Tonic current in this given CA1 PC is 410.19 − 351.95 = 58.24 pA.

Whole-cell voltage-clamp recordings were performed in naïve control (NC), artificial cerebrospinal fluid (ACSF) -injected and Aβ_1-42_-injected mice in the str. pyramidale of the CA1 hippocampal region (Figure 1). Application of 100 µM of bicuculline methiodide (BMI), along with the inhibitory postsynaptic currents (IPSC) blockade, resulted in tonic inhibition in the CA1 pyramidal cells measured as a shift in the holding current [16]. Mice injected with Aβ_1-42_ were found to exhibit a significant increase in tonic inhibition in comparison to NC (78.070 ± 19.950 pA versus 50.948 ± 9.263 pA, *p* = 0.0184) and artificial cerebrospinal fluid (ACSF)-injected (78.070 ± 19.950 pA versus 35.051 ± 5.340 pA, *p* = 0.0015) mice (Figure 1). Aβ_1-42_ did not induce changes either in the input resistance (NC = 150.20 ± 48.53 MΩ, ACSF-injected = 170.70 ± 52.57 MΩ, Aβ_1-42_-injected = 115.70 ± 35.63 MΩ), or the membrane capacitance of the cell (NC = 76.27 ± 17.75 pF, ACSF-injected = 62.39 ± 17.75 pF, Aβ_1-42_-injected = 83.43 ± 18.25 pF). Likewise, no changes were found between groups for the series resistance (NC = 21.15 ± 4.21 MΩ, ACSF-injected = 19.07 ± 6.804 MΩ, Aβ_1-42_-injected = 20.32 ± 6.04 MΩ).

Similar to our observations, increased tonic conductance has also been observed previously in the dentate gyrus (DG) region of a Tg 5xFAD and APP/PS1 AD mouse model, in which it was associated with cognitive decline [6,7]. Overall, the findings from these AD mouse models imply that excessive amounts of GABA in the extracellular space is potentially released from reactive astrocytes, and reduction of tonic inhibition in these mice rescues long-term potentiation (LTP) impairment and memory decline [6,7]. It is important to point out that, other potential factors might also contribute to the altered extrasynaptic GABA levels, from changes in GABA uptake and clearance processes. Furthermore, remodeling of GABA_A_R subunit expression, along with alteration of the expression of GABA transporters (GATs) in the AD brain, may buffer changes in synaptic GABA levels, and ultimately, affect the total levels of extrasynaptic GABA [16,17,18,19,20].

**Figure 2:**
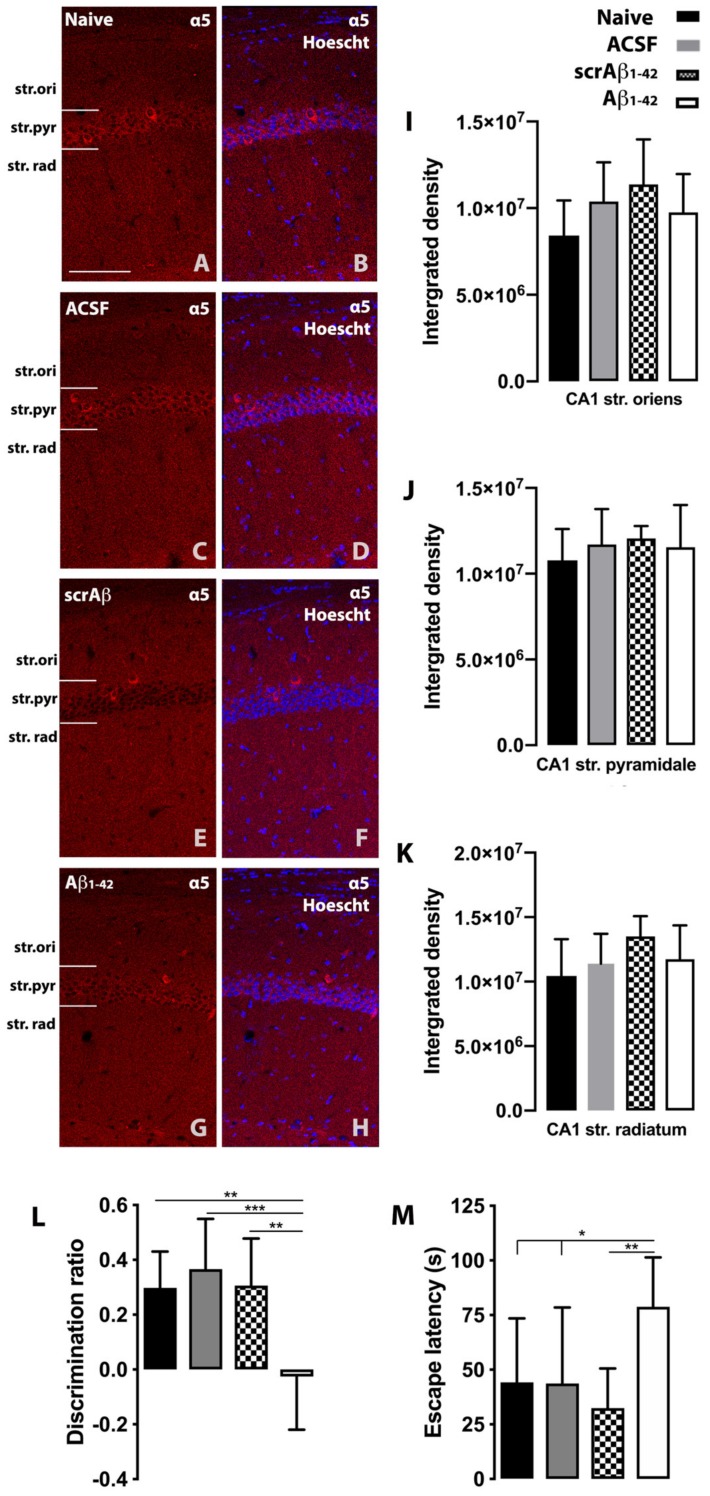
(**A**–**H**) Representative photomicrographs of the hippocampal CA1 region showing GABA_A_R *α*5 subunit expression (red) and *α*5 immunoreactivity overlaid with Hoechst (blue) labeling for representative naïve control, artificial cerebrospinal fluid- (ACSF), scrambled Aβ_1-42_- (scrAβ_1-42_) and Aβ_1−42_-injected mice (Aβ_1−42_). (**I**–**K**)**:** Quantification of GABA_A_R *α*5 subunit immunoreactivity in the regions and layers of the hippocampus in NC, ACSF-, scrAβ_1-42_- and Aβ_1-42_-injected mice. Scale bar, 50 *μ*m. (**L,M**) Aβ_1−42_-injected mice show long- and short-term spatial memory impairment revealed by the novel object recognition (**L**) and Water Morris Maze (**M**) tests, respectively. The Aβ_1-42_-lesioned mice do not discriminate between novel and familiar objects and show longer escape latency in trial 4 of the MWM task. Data are expressed as mean ± SD (one-way ANOVA and Tukey’s post-hoc test (* *p* < 0.05, ** *p* < 0.001, *** *p* < 0.001; NC *n* = 9, ACSF-injected *n* = 9, scrAβ_1-42_-injected *n* = 9 and Aβ_1-42_-injected *n* = 9).

We measured tonic inhibition in the CA1 region of Aβ_1-42_ treated mice as the CA1 region has been reported to be one of the earliest hippocampal regions to exhibit functional changes in AD [21]. The hippocampus might have discrete functional domains along its regions and subregions that affect learning and memory processes, so region-specific differences could also be observed in the deterioration of the LTP during AD progression. Several studies have reported differences in LTP induction and amplitude within the dorsal and ventral parts of the CA1 region in rodents [22]. While some studies using Tg AD mouse models [6,7] have indicated the relevance of DG-related impaired LTP and memory deficits in the AD rodent brain, our data indicate that the CA1 region might be, at least, equally critical in the process of the memory decline in AD. Increased tonic inhibition after Aβ injection might be a consequence of increased extracellular GABA levels that lead to the overactivation of α5 subunit containing receptors in the CA1 pyramidal cells of the hippocampus. We have also considered the possibility of α5 subunit upregulation as a contributing factor to increased tonic inhibition. However, our results show no Aβ_1-42_ induced alterations in α5 subunit expression (Figure 2A–K). Previous data suggest that the α5 subunit containing GABA_A_ receptor plays a key role in cognition, and it potentially represents an important component of synaptic transmission in the CA1 region of the hippocampus, which might be impaired by the action of the Aβ protein fragment [23]. The enhanced performance of α5-deficient mice in learning tasks was linked to decreased GABA-mediated synaptic inhibition in the hippocampus, as shown by the reduced peak amplitudes and slower decay times of IPSCs [24]. Increased tonic GABAergic inhibition can alter E/I balance in the hippocampus, which potentially contributes to the cognitive decline observed in Aβ_1-42_-treated mice. At day 7 post-injection, Aβ_1−42_-injected mice showed significant spatial memory impairment compared with NC (*p* = 0.002 and *p* = 0.0481), ACSF-injected (*p* = 0.0003 and *p* = 0.0445) and scrambled Aβ_1-42_ (scrAβ_1-42_)-injected (*p* = 0.0015 and *p* = 0.005) mice in the novel object recognition (NOR) and Water Morris Maze (MWM) tasks (Figure 2L,M). The significantly lower discrimination ratio found in Aβ_1−42_-injected mice compared with ACSF-injected and NC mice, indicates that Aβ_1−42_-injected mice could not discriminate between familiar and novel objects (Figure 2L). Aβ_1−42_-injected mice also show an increased escape latency to find the platform in the MWM (Figure 2M).

Tonic inhibition is relevant not only for the control of pyramidal cell excitability in the hippocampus, but also for the control of oscillatory network activity mediated by interneurons. Interneurons from the CA1 region of the hippocampus exhibit tonic currents that reduce their excitability, thus limiting their inhibitory output to excitatory pyramidal cells [25]. Fast-spiking PV positive basket cell interneurons that create perisomatic synapses with excitatory pyramidal neurons mediate network oscillations in the hippocampus [26,27], and this is critical for learning and memory processes in the hippocampus. Interestingly, these interneurons display a dual function, which is modulated by extracellular GABA levels. At low extracellular GABA levels, extrasynaptic GABA_A_Rs induce depolarization in these interneurons, which facilitates the transmission of inhibitory signals to the excitatory pyramidal cells. However, under pathological conditions, such as with the accumulation of Aβ_1-42_, increased extracellular GABA levels would induce hyperpolarization in the interneurons [28]. Furthermore, the aforementioned tonic inhibition-dependent effects in the hippocampal circuitry would manifest as a loss of oscillatory and rhythmic hippocampal activity [16,29]. Thus, increased extrasynaptic tonic inhibition in the hippocampus would affect interneuron function by altering communication with other interneurons and with principal pyramidal cells. In turn, this would affect the modulation of hippocampal excitability and the maintenance of a normal neuronal network.

Much research has effectively shown that neuronal loss, and the resulting brain atrophy, might be one of the leading factors associated with cognitive impairment in AD [30]. However, other neuropathological processes are likely to contribute to the dysfunctional E/I network balance in the hippocampus that leads to cognitive deterioration. Although cognitive decline in AD has been largely related to Aβ oligomers and the glutamatergic system through multiple interactions [31,32], there is growing evidence to suggest that cognitive decline in the AD brain is influenced and also partly precipitated by altered GABAergic function [19], and this has been linked to cognitive decline [33]. In the extrasynaptic space, Aβ_1-42_ mediates increase in ambient GABA levels which chronically activates tonic inhibitory currents in the hippocampus, that are involved in the maintenance of the excitatory network and synchronization, and in turn might contribute to the cognitive deficits - but the exact mechanism requires further elucidation.

In summary, we demonstrated that tonic GABAergic current is increased in Aβ_1-42_-injected mice, suggesting that, along with the classic features of AD that contribute to the cognitive decline, such as neuronal loss, tau and vascular pathology and neuroinflammation, increased GABAergic tonic inhibitory currents may also play a key role in hippocampal deficits in AD. 

## 3. Materials and Methods 

### 3.1. Animals

The animals were housed at the University of Auckland, Vernon Jensen Unit and the University of Otago, Hercus Taieri Resource Unit under 12 h reverse light-cycle conditions (lights on at 8 PM), with ad libitum access to food and water. All experiments were conducted in accordance with the National Animal Ethics Advisory Committee guidelines and the approval of the institutional animal ethics committee of the University of Auckland and University of Otago (Approval numbers: 60/13, 001586 and 001655). The animals were maintained under conditions of a 12 h light/dark cycle (lights on at 7:00 P.M.) with food and water available ad libitum. Experiments were performed on young 2 months old C57BL/6 wild-type male mice (whole-cell voltage-clamp recording, *n* = 4–5; behavioural testing and immunohistochemistry, *n* = 9).

### 3.2. Aβ_1-42_ Preparation

Aβ_1-42_ is routinely produced as a recombinant protein fused with maltose-binding protein (MBP), with a proteolytic cleavage site for Factor X protease between the two segments based on (Wilson C. MSc Thesis, University of Otago, 2007) [34,35]. This strategy utilizes the solubilizing character of the maltose-binding protein, a product of the MalE gene, to ensure expression of the soluble protein at a high concentration in *Escherichia coli*. After the expression of this recombinant fusion protein in bacteria, the product was purified on an amylose column to which the MBP segment of the protein binds. The affinity selected fusion protein was eluted from the resin with maltose and concentrated by ammonium sulphate precipitation. The carrier MBP was then cleaved off the fusion protein by Factor X protease, and the released Aβ_1-42_ was isolated and further purified by hydrophobic chromatography in 0–50% *v*/*v* acetonitrile/0.1% *v*/*v* trifluoroacetic acid (TFA), using fast protein liquid chromatography (FPLC). The fractions containing pure Aβ_1-42_ were detected immunologically with an antibody against residues 17–24 of Aβ_1-42_ and lyophilized to remove the solvent. Mass spectrometry was used to confirm the expected molecular ion for the desired product. The concentration of the protein fragment has been determined by bicinchoninic acid assay (BCA) at 60 °C for 30 min. Incubating the BCA assay at higher temperatures is recommended as a way to increase assay sensitivity while minimizing the variances caused by unequal amino acid composition. Before intra-hippocampal injection of this product, we diluted the prepared monomer in ACSF (147 mM Na^+^, 3.5 mM K^+^, 2 mM Ca^2+^, 1 mM Mg^2+^ [pH 7.3]) and ‘aged’ the solution at 37 °C for 48 h to facilitate the formation of soluble aggregates, which was confirmed by SDS/PAGE and by non-dissociating PAGE. For the non dissociating gel, the separating gel was a 12.5% (*w*/*v*) acrylamide gel (buffer 750 mM Tris-HCL pH 8.8), stacking gel 9% (*w*/*v*) acrylamide (buffer 125 mM Tris- HCL pH 6.8) with the running buffer 25 mM Tris and 19.2 mM glycine. Electrophoresis was at 200 V for 1.5 h. 

The optimal incubation time required to produce the highly toxic oligomers is 48–120 h. Western blots of an ageing profile of Aβ_1-42_ were analyzed both on non-dissociating gels, where the monomer decreases, and an oligomer appears by 48 h, and SDS gels where the dimer and trimer of Aβ_1-42_ are seen as well as a higher molecular weight oligomer [35]. Only aggregates from misfolded Aβ_1-42_ are deduced to be SDS insoluble [36], explaining why SDS gels show lower amounts with the less stably-aggregated species dissociated by the SDS [35]. Furthermore, it is very likely that the aged amyloid beta sample injected that included dimers, trimers and possible tetramers underwent further aggregation in the extracellular space following intracerebral injection. Importantly, there are several studies that provide evidence for the existence of toxic oligomeric Aβ species in the AD brain but there is controversy and unanswered questions regarding their precise structures, spread, amplification and how they induce neuronal dysfunction and death [37].

### 3.3. Aβ_1-42_ Stereotaxic Injection

Mice were anesthetized by subcutaneous injection of 75 mg/kg ketamine and 1 mg/kg domitor. Bilateral coordinates for stereotaxic Aβ_1-42_ injection at three depths within the CA1 region of the hippocampus were determined relative to bregma (antero-posterior, −2.0 mm; medial-lateral, ±1.3 mm; dorsal-ventral, −1.9, 2.4, and 2.9 mm) according to the Paxinos and Franklin’s mouse brain atlas [38]. Stereotaxic bilateral administration of 1 μL of 20 μM neurotoxic Aβ_1-42_ [34,35] or ACSF as vehicle or scrAβ_1-42_ (AS-25382, AnaSpec) into the CA1 region was performed at a rate of 0.1 µL/min. 

### 3.4. Whole-Cell Voltage-Clamp Recording 

#### 3.4.1. Slice Preparation 

Mice from the NC, ACSF-injected and Aβ_1-42_-injected groups were euthanized by cervical dislocation and decapitated at 7 days after Aβ_1-42_ injection. The brain was rapidly placed in protective ice-cold ACSF containing (mM): N-methyl-D-glucamine (NMDG) (93), KCl (93), NaH_2_PO_4_ (1.2), NaHCO_3_ (30), HEPES (20), D-glucose (25), sodium ascorbate (5), thiourea (2), sodium pyruvate (3), MgSO_4_ (10) and CaCl_2_ (0.5), pH 7.35. Hippocampal slices were cut at 350 µm using a vibratome (Leica VT 12000, Wetzlar, Germany). The slices were incubated in a chamber (GD100, Grant, Cambridge, UK) containing protective ACSF at 34 °C for 12 min and transferred and stored for recovery at 21 °C in a holding chamber in ACSF recording solution containing (mM): NaCl (97), KCl (2.5), NaH_2_PO_4_ (1.2), NaHCO_3_ (30), HEPES (20), D-glucose (25), sodium ascorbate (5), thiourea (2), sodium pyruvate (3), MgSO_4_ (2) and CaCl_2_ (2) pH 7.35, in the presence of 3 mM kynurenic acid (K3375, Sigma Aldrich, St. Louis, MO, USA) and 5 µM GABA (A2129, Sigma Aldrich).

#### 3.4.2. Whole-Cell Patch-Clamp Recording

CA1 pyramidal cells were visualized under IR-DIC optics (Olympus TH4-200, BX51WI, Tokyo, Japan) equipped with a video camera (CCD, C7500-51, S.No 6 × 0047; Hamamatsu, Hamamatsu, Japan). Voltage-clamp recordings were performed using a MultiClamp 700B amplifier (Axon Instruments, San Jose, CA, USA). Slices were continuously perfused with ACSF saturated with 95% O_2_/5% CO_2_, containing 3 mM kynurenic acid and 5 µM GABA. Glass microelectrodes (resistance 3–5 MΩ) were created using a dual-stage glass micropipette puller PC-10 (Narishige, Tokyo, Japan) and were filled with the following internal solution (mM): CsCl (140), MgCl_2_ (1), HEPES (10), EGTA (0.1), NaCl (4), MgATP (2), NaGTP (0.3) pH 7.28, (osmolarity, ~280 mOsm). Neurons were held at −70 mV in voltage-clamp configuration. Recordings were discarded if series resistance altered more than 30% during the duration of the recording. After obtaining a stable 5 min baseline recording, defined as a lack of change in the holding current, drugs were perfused and tonic conductance was recorded.

Before recording, hippocampal slices were pre-incubated in ACSF with 5 μM GABA to increase the ambient GABA levels, so as to facilitate the measurement of tonic conductance, as well as with 3 mM kynurenic acid to block ionotropic glutamate receptors. Tetrodotoxin (TTX) (1078, Tocris, Bristol, UK) at 5 µM and CdCl_2_ (202908, Sigma Aldrich) at 50 µM were used to block voltage-gated Na^+^ and Ca^++^ channels, respectively. Finally, the GABA receptor antagonist BMI was used at 100 µM to measure tonic conductance. All recordings were low-pass filtered at 3 kHz and digitized at 10 Hz using an Axon Digidata 1550B plus HumSilencer apparatus (Axon Instruments, San Jose, CA, USA and SDR Scientific, Sydney, NSW, Australia). 

#### 3.4.3. Tonic Current Measurements in Hippocampal Slices

The tonic current was measured based on the shift in the holding current caused by the addition of the GABA_A_ receptor antagonist BMI. Tonic current measurement was performed following the method described by [16]. Briefly, tonic current was defined by generating all-points histograms for the control and the BMI periods, and later, a Gaussian curve was fit to the data. Tonic inhibition was determined by subtracting the mean current value for the control period from the mean current value for the BMI period.

### 3.5. Behavioral Testing

Behavioral testing was performed to elucidate the effects of Aβ_1−42_ on the cognitive performance of the mice using the NOR and MWM tasks. On day 6 and 7 after Aβ_1−42_ injection the NOR test was started at 9 AM and on day 7 the MWM test at 11 AM. The behavioral analysis was performed using the tracking image analyzer system EthoVision XT 9 (Noldus; Wageningen, Netherlands). 

#### 3.5.1. Novel Object Recognition Test

To evaluate long-term spatial memory, the NOR test was performed in a square arena with non-transparent plexiglass walls (25 cm × 29 cm × 25 cm). Each mouse was placed in the arena individually and given 10 min to habituate to the environment. Next, two identical objects were introduced in the arena at designated locations, and the mice were given 5 min to interact with and explore the objects. The following day (24 h later), the animals were presented with a similar set of objects but one object was novel to them; they were allowed to freely explore the objects again for a 5 min period. The amount of time spent to explore the new object is considered as an index of recognition memory. The discrimination ratio for a novel over a familiar object was calculated as follows: time near a new object minus the time near the old object, divided by time near the new object plus the time near the old object [34,35]. 

#### 3.5.2. Morris Water Maze Test

The MWM task was used to assess short-term spatial memory. The MWM apparatus comprises a circular black tank (diameter, 130 cm; height, 130 cm) filled with tap water. A constant temperature of 20 °C was maintained during every test. A circular escape platform of 10 cm diameter and several navigation cues were used to provide spatial orientation for the mice. A video camera suspended from the ceiling was used to record every test. The starting position of every mouse was assigned randomly. The location of the hidden platform was kept constant. If the mouse did not find the hidden platform within 90 s, the animal was placed on the platform for 10 s before being returned to the cage. Spatial learning was tested across four repeat trials on day 7 after Aβ_1−42_ injection. Between trials, mice were dried with a towel, placed in their cages and kept over a heating blanket for 10 min. The latency period before the mouse reached the platform was measured. 

### 3.6. Tissue Processing and Immunohistochemistry

Following behavioral testing, for immunohistochemistry experiments mice were deeply anesthetized with 75 mg/kg ketamine and 1 mg/kg domitor (Pfizer; New York, NY, USA) and perfused transcardially with 20 mL of ice-cold 4% paraformaldehyde in phosphate buffer, pH 7.6. Brains were removed and post-fixed in paraformaldehyde solution for 2 h at 21 °C and then incubated in 30% sucrose in Tris-phosphate saline (TBS; pH 7.6, 0.05 M Tris, 0.15 M NaCl) solution overnight at 4 °C. Four serial sets of 30 μm-thick coronal brain sections were cut using a freezing microtome [34,35,39]. 

Free-floating single-label fluorescence immunohistochemistry was performed to detect the GABA_A_R α5 subunit within the mouse hippocampus [34]. Two hippocampal brain sections from each animal were first incubated in blocking solution containing 0.25% bovine serum albumin, 0.3% Triton X-100, and 1% donkey serum in TBS for 1 h at 21 °C followed by incubation with the primary antibody (1:1000, Thermo Fisher Scientific, PA5-31163, Waltham, MA, USA) for 48 h at 4 °C. The sections were then incubated in donkey anti-rabbit Alexa 647 (1:500; ThermoFisher Scientific) for 2 h at 21 °C. Nuclei were counterstained with Hoechst 33,342 (1:10,000; Invitrogen, Carlsbad, CA, USA). Sections were then mounted on slides, air dried overnight, and coverslipped with Mowiol mounting medium. Omission of primary antibodies resulted in a complete absence of immunoreactivity. The experimenter was blinded to avoid any potential bias during image acquisition and analysis. Imaging was conducted using a Zeiss 710 confocal laser-scanning microscope (Carl Zeiss; Oberkochen, Germany) as described in [39]. Integrated density measurements were undertaken using ImageJ, with the size of the measured areas as follows: 21,352 μm^2^ for the CA1 region, 4761 μm^2^ for the CA3 region, and 12,295 μm^2^ for the DG in each layer. Intensity measurements were taken in the regions of the stratum (str.) pyramidale, str. radiatum, and str. moleculare of the CA1 region.

### 3.7. Statistical Analysis 

All data are expressed as mean ± SD. One-way ANOVA followed by Tukey *post-hoc* test was used to examine differences between the different groups in all experiments. All statistical analyses were conducted using Prism (version 8, GraphPad), and a *p* < 0.05 was considered to indicate statistical significance.

## References

[1] Pakaski M., Farkas Z., Kasa P., Forgon M., Papp H., Zarandi M., Penke B., Kasa P. (1998). Vulnerability of small GABAergic neurons to human beta-amyloid pentapeptide. Brain Res..

[2] Gao R., Penzes P. (2015). Common mechanisms of excitatory and inhibitory imbalance in schizophrenia and autism spectrum disorders. Curr. Mol. Med..

[3] Ren S.-Q., Yao W., Yan J.-Z., Jin C., Yin J.-J., Yuan J., Yu S., Cheng Z. (2018). Amyloid β causes excitation/inhibition imbalance through dopamine receptor 1-dependent disruption of fast-spiking GABAergic input in anterior cingulate cortex. Sci. Rep..

[4] Li Y., Sun H., Chen Z., Xu H., Bu G., Zheng H. (2016). Implications of GABAergic Neurotransmission in Alzheimer’s Disease. Front Aging Neurosci..

[5] Madl J.E., Royer S.M. (2000). Glutamate dependence of GABA levels in neurons of hypoxic and hypoglycemic rat hippocampal slices. Neuroscience.

[6] Wu Z., Guo Z., Gearing M., Chen G. (2014). Tonic inhibition in dentate gyrus impairs long-term potentiation and memory in an Alzheimer’s disease model. Nat. Commun..

[7] Jo S., Yarishkin O., Hwang Y.J., Chun Y.E., Park M., Woo D.H., Bae J.Y., Kim T., Lee J., Chun H. (2014). GABA from reactive astrocytes impairs memory in mouse models of Alzheimer’s disease. Nat. Med..

[8] Crestani F., Keist R., Fritschy J.M., Benke D., Vogt K., Prut L., Bluthmann H., Mohler H., Rudolph U. (2002). Trace fear conditioning involves hippocampal alpha5 GABA(A) receptors. Proc. Natl. Acad. Sci. USA.

[9] Martin L., Oh G., Orser B. (2009). Etomidate targets alpha5 gamma-aminobutyric acid subtype A receptors to regulate synaptic plasticity and memory blockade. Anesthesiology.

[10] Limon A., Reyes-Ruiz J.M., Miledi R. (2011). GABAergic drugs and Alzheimer’s disease. Future Med. Chem..

[11] Louzada P., Lima A., Mendonca-Silva D., Noël F., de Mello F., Ferreira S. (2004). Taurine prevents the neurotoxicity of β-amyloid and glutamate receptor agonists: Activation of GABA receptors and possible implications for Alzheimer’s disease and other neurological disorders. Faseb. J..

[12] Sanchez P.E., Zhu L., Verret L., Vossel K.A., Orr A.G., Cirrito J.R., Devidze N., Ho K., Yu G.-Q., Palop J.J. (2012). Levetiracetam suppresses neuronal network dysfunction and reverses synaptic and cognitive deficits in an Alzheimer’s disease model. P. Natl. Acad. Sci..

[13] Marcade M., Bourdin J., Loiseau N., Peillon H., Rayer A., Drouin D., Schweighoffer F., Désiré L. (2008). Etazolate, a neuroprotective drug linking GABAA receptor pharmacology to amyloid precursor protein processing. J. Neurochem..

[14] Vellas B., Sol O., Snyder P.J., Ousset P.J., Haddad R., Maurin M., Lemarie J.C., Desire L., Pando M.P. (2011). EHT0202 in Alzheimer’s disease: A 3-month, randomized, placebo-controlled, double-blind study. Curr. Alzheimer Res..

[15] Calvo-Flores Guzman B., Vinnakota C., Govindpani K., Waldvogel H.J., Faull R.L.M., Kwakowsky A. (2018). The GABAergic system as a therapeutic target for Alzheimer’s disease. J. Neurochem..

[16] Glykys J., Mody I. (2006). Hippocampal network hyperactivity after selective reduction of tonic inhibition in GABA A receptor alpha5 subunit-deficient mice. J. Neurophysiol..

[17] Marczynski T.J. (1998). GABAergic deafferentation hypothesis of brain aging and Alzheimer’s disease revisited. Brain Res. Bull..

[18] Fuhrer T.E., Palpagama T.H., Waldvogel H.J., Synek B.J.L., Turner C., Faull R.L., Kwakowsky A.L. (2017). Impaired expression of GABA transporters in the human Alzheimer’s disease hippocampus, subiculum, entorhinal cortex and superior temporal gyrus. Neuroscience.

[19] Govindpani K., Calvo-Flores Guzman B., Vinnakota C., Waldvogel H.J., Faull R.L., Kwakowsky A. (2017). Towards a Better Understanding of GABAergic Remodeling in Alzheimer’s Disease. Int. J. Mol. Sci..

[20] Kwakowsky A., Calvo-Flores Guzmán B., Govindpani K., Waldvogel H.J., Faull R.L. (2018). Gamma-aminobutyric acid A receptors in Alzheimer’s disease: Highly localized remodeling of a complex and diverse signaling pathway. Neural Regen. Res..

[21] Kwakowsky A., Calvo-Flores Guzman B., Pandya M., Turner C., Waldvogel H.J., Faull R.L. (2018). GABAA receptor subunit expression changes in the human Alzheimer’s disease hippocampus, subiculum, entorhinal cortex and superior temporal gyrus. J. Neurochem..

[22] Masurkar A.V. (2018). Towards a circuit-level understanding of hippocampal CA1 dysfunction in Alzheimer’s disease across anatomical axes. J. Alzheimers Dis. Parkinsonism.

[23] Maruki K., Izaki Y., Nomura M., Yamauchi T. (2001). Differences in paired-pulse facilitation and long-term potentiation between dorsal and ventral CA1 regions in anesthetized rats. Hippocampus.

[24] Collinson N., Kuenzi F.M., Jarolimek W., Maubach K.A., Cothliff R., Sur C., Smith A., Otu F.M., Howell O., Atack J.R. (2002). Enhanced learning and memory and altered GABAergic synaptic transmission in mice lacking the alpha 5 subunit of the GABAA receptor. J. Neurosci..

[25] Semyanov A., Walker M.C., Kullmann D.M. (2003). GABA uptake regulates cortical excitability via cell type–specific tonic inhibition. Nat. neurosci..

[26] Bartos M., Vida I., Jonas P. (2007). Synaptic mechanisms of synchronized gamma oscillations in inhibitory interneuron networks. Nat. rev. neurosci..

[27] Mann E.O., Paulsen O. (2007). Role of GABAergic inhibition in hippocampal network oscillations. Trends Neurosci..

[28] Song J., Zhong C., Bonaguidi M.A., Sun G.J., Hsu D., Gu Y., Meletis K., Huang Z.J., Ge S., Enikolopov G. (2012). Neuronal circuitry mechanism regulating adult quiescent neural stem-cell fate decision. Nature.

[29] Lee V., Maguire J. (2014). The impact of tonic GABAA receptor-mediated inhibition on neuronal excitability varies across brain region and cell type. Front Neural Circuits.

[30] Fox N.C., Scahill R.I., Crum W.R., Rossor M.N. (1999). Correlation between rates of brain atrophy and cognitive decline in AD. Neurology.

[31] Lauren J., Gimbel D.A., Nygaard H.B., Gilbert J.W., Strittmatter S.M. (2009). Cellular prion protein mediates impairment of synaptic plasticity by amyloid-beta oligomers. Nature.

[32] Revett T., Baker G., Jhamandas J., Kar S. (2013). Glutamate system, amyloid β peptides and tau protein: Functional interrelationships and relevance to Alzheimer disease pathology. J. Psychiatry Neurosci..

[33] Ramos-Miguel A., Hercher C., Beasley C.L., Barr A.M., Bayer T.A., Falkai P., Leurgans S.E., Schneider J.A., Bennett D.A., Honer W.G. (2015). Loss of Munc18-1 long splice variant in GABAergic terminals is associated with cognitive decline and increased risk of dementia in a community sample. Mol. Neurodegener..

[34] Kwakowsky A., Potapov K., Kim S., Peppercorn K., Tate W., Ábrahám I. (2016). Treatment of beta amyloid 1-42(Aβ_1-42_)-induced basal forebrain cholinergic damage by a non-classical estrogen signaling activator in vivo. Sci. Rep..

[35] Yeung J., Tate W., Palpagama T., Peppercorn K., Waldvogel H., Faull R.L., Kwakowsky A. (2020). The Acute Effects of Amyloid-Beta1−42 on Glutamatergic Receptor and Transporter Expression in the Mouse Hippocampus. Front. Neurosci..

[36] Hillen H. (2019). The Beta Amyloid Dysfunction (BAD) Hypothesis for Alzheimer’s Disease. Front. Neurosci..

[37] Chen X.-Q., Mobley W.C. (2019). lzheimer Disease Pathogenesis: Insights From Molecular and Cellular Biology Studies of Oligomeric Aβ and Tau Species. Front Neurosci..

[38] Paxinos G., Franklin K. (2000). The Mouse Brain in Stereotaxic Coordinates.

[39] Palpagama T., Sagniez M., Kim S.H., Waldvogel H.J., Faull R.L., Kwakowsky A. (2019). GABAA receptors are well preserved in the hippocampus of aged mice. eNeuro.

